# Can Botulinum Toxin Type A effectively treat neuropathic pain in spinal cord injury? A protocol of systematic review and meta-analysis

**DOI:** 10.1097/MD.0000000000020702

**Published:** 2020-06-19

**Authors:** Zeng-mian Wang, Ze-yu Wang, Chun-jie Wei, Yao-jia Jiang

**Affiliations:** Third Ward of Neurology Department, First Affiliated Hospital of Jiamusi University, Jiamusi, China.

**Keywords:** botulinum toxin type A, efficacy, neuropathic pain, randomized controlled trial, safety, spinal cord injury

## Abstract

**Background:**

This protocol aims to assess the efficacy and safety of Botulinum Toxin Type A (BTTA) for the treatment of neuropathic pain (NPP) in patients with spinal cord injury (SCI).

**Methods:**

We will retrieve databases in Cochrane Library, EMBASE, MEDLINE, Excerpta Medica Database, PsychINFO, the Allied and Complementary Medicine Database, and Chinese Biomedical Literature Database, and China National Knowledge Infrastructure from the beginning to the March 1, 2020. We will consider any potential studies on assessing the efficacy and safety of BTTA for the treatment of NPP in patients with SCI without limitations of language and publication status. Cochrane risk of bias will be used to assess the risk of bias for all included trials. RevMan 5.3 software will be utilized to synthesize the extracted data and to analyze those data.

**Results:**

This study will appraise the efficacy and safety based on the current evidence of BTTA for the treatment of NPP in patients with SCI.

**Conclusion:**

This study will exert high quality clinical trials for exploring the efficacy and safety of BTTA in treating NPP in patients with SCI.

**PROSPERO registration number:**

PROSPERO CRD42020170474.

## 1 Introduction

Neuropathic pain (NP) is 1 of the most common complications in patients with spinal cord injury (SCI).^[1–3]^ Studies reported that the prevalence of pain in SCI patients varies from 75% to 81%,^[4–6]^ and NP accounts for 53% of all SCI pain patients,^[7]^ which significantly reduce quality of life in such patients.^[8–9]^

Botulinum Toxin Type A (BTTA) is commonly applied to treat spasticity or dystonia.^[10–13]^ Recent studies have suggested that it is effective for the management of NP following SCI.^[14–21]^ To date, there is not yet a synthesis of current studies that investigated the efficacy and safety of BTTA for the treatment of neuropathic pain (NPP) after SCI. This is the first study to evaluate the efficacy and safety of BTTA for the treatment of NPP following SCI systematically and comprehensively.

## 2 Methods

### 2.1 Study registration

This study has been registered on PROSPERO (CRD42020170474). It follows the guideline of Preferred Reporting Items for Systematic Reviews and Meta-Analysis Protocol statement guidelines.^[22]^

### 2.2 Study selection criteria

#### 2.2.1 Study types

This study will include randomized controlled trials (RCTs) alone that assessing the efficacy and of BTTA for the treatment of NPP in patients with SCI without restrictions of language and publication status. Any other studies, such as laboratory studies, letters, reviews, case reports, uncontrolled trials, and non-RCTs will be removed.

#### 2.2.2 Interventions

All patients in the experimental group received BTTA alone for the treatment of their NPP condition. The combined therapy of BTTA with other interventions will be excluded.

All participants in the control group underwent any therapies for the NPP, but not any types of BTTA.

#### 2.2.3 Population

Any SCI participants who were diagnosed as NPP will be included. All information relating to the race, age, gender, or economic status will not be taken into account.

#### 2.2.4 Outcomes

The primary outcome includes the pain intensity of NPP, as measured by any validated pain scales, such as NPP Symptom Inventory.

The secondary outcomes consist of spasticity (as assessed by Modified Ashworth Scale or any relevant scales), sleep quality (as identified by The Pittsburgh Sleep Quality Index or other related scores), depression and anxiety (as evaluated by Hamilton Depression Rating Scale and the Hamilton Anxiety Rating Scale, or other associated tools), quality of life (as investigated by World Health Organization quality of life or any connected questionnaires), and adverse events.

#### 2.2.5 Search strategy

The following electronic databases will be searched: Cochrane Library, EMBASE, MEDLINE, Excerpta Medica Database, PsychINFO, the Allied and Complementary Medicine Database, and Chinese Biomedical Literature Database, and China National Knowledge Infrastructure from the initial to the March 1, 2020. We will include RCTs alone that exploring the efficacy and safety of BTTA for the treatment of NPP in patients with SCI. No limitations of language and publication status will be implied.

The sample of search strategy for Cochrane Library is listed in Table 1. We will adapt similar search strategies for other electronic databases.

**Table 1:**
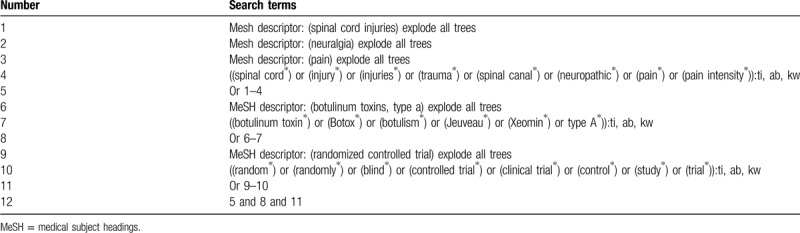
Search strategy applied in Cochrane library database.

Additionally, websites of clinical trial registry and reference lists of associated reviews will be searched.

#### 2.2.6 Study selection

**Figure 1:**
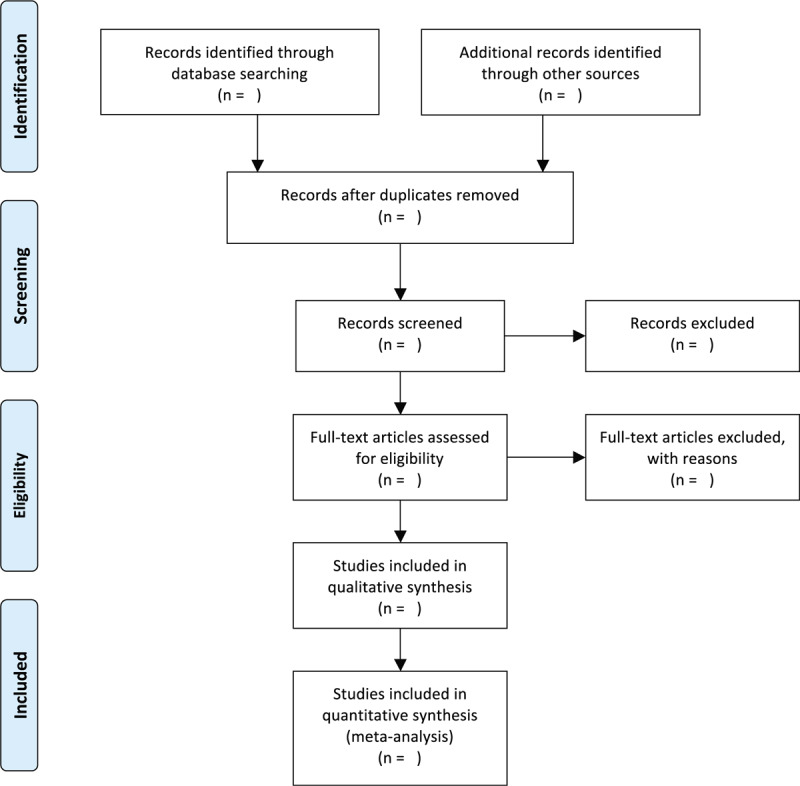
Flowchart of study selection.

Two authors will independently read the titles/abstracts of search literatures based on the predefined eligibility criteria. All irrelevant records will be removed after initial screen. Then, full-papers of all potential trials will be identified carefully against all inclusion criteria. Any conflicts between 2 authors will be solved by a third author through consultation. The whole process of study selection will be shown in a flow chart (Fig. 1).

#### 2.2.7 Data extraction and management

Two authors will separately extract data from included RCTs using predefined data extraction sheet. The extracted information is as follows: first author, year of publication, subject information (such as age, gender, disease duration), trial setting, trial methods, sample size, interventions, controls, outcome indicators, adverse events, conflicts of interest, and any other relevant information. If there are disagreements between 2 authors, they will be resolved by consulting and discussing with the help of another experienced author.

#### 2.2.8 Risk of bias assessment

Cochrane Risk of Bias Tool will be used for the assessment of study quality through 7 domains. Each 1 is divided into 3 types: high risk of bias, unclear risk of bias, and low risk of bias. Any discrepancies in the process will be settled down with the help of another experienced author by discussion.

#### 2.2.9 Measurement of treatment effect

For continuous variables, mean difference or standardized mean difference and 95% confidence intervals will be used to calculate them. For dichotomous variables, risk ratio and 95% confidence intervals will be utilized to present them.

#### 2.2.10 Unit of analysis

If the eligible trials belong to the cross-over studies, we will assess the first period of study data only.

#### 2.2.11 Missing data

If the data in the eligible trials is missing or insufficient, we will contact primary authors to request it. If we can not receive such data, we will use intent-to-treat analysis for data analysis based on the available data.

#### 2.2.12 Assessment of heterogeneity

In this study, *I*^2^ test will be applied to check heterogeneity among the included trials. When *I*^2^ ≤50%, we will consider heterogeneity as reasonable, and will we carry out a fixed-effects model for outcome data pooling. When *I*^2^ >50%, we will regard heterogeneity as obvious, and we will perform a random-effects model for the outcome data synthesizing.

#### 2.2.13 Data synthesis

We will use RevMan 5.3 software to carry out statistical analysis. If reasonable heterogeneity is found across sufficient trials, we will perform a meta-analysis based on the similar study information, patient characteristics, interventions, controls and outcome indicators. Otherwise, we will perform subgroup analysis to explore potential sources of significant heterogeneity. If it is not possible to conduct a meta-analysis, we will synthesize outcome data using a narrative summary. It will be reported by detailed written commentary to demonstrate the findings, target participant characteristics, intervention and controls (such as BTTA vs acupuncture), and types of outcome measurements (such as pain intensity of NPP, spasticity, sleep quality, or incidence of adverse events).

#### 2.2.14 Subgroup analysis

If data are available, we will carry out a subgroup analysis according to the different types of interventions, comparators, outcome indicators, and study quality.

#### 2.2.15 Sensitivity analysis

If necessary, we will conduct sensitivity analysis to recognize the stability of study finding by crossing out low quality studies.

#### 2.2.16 Publication bias

If at least 10 trials are included in this study, we will detect potential reporting bias using funnel plot,^[23]^ Egger regression and Begger tests.^[24]^

## 3 Discussion

Although several previous trials have addressed that BTTA is used for the treatment of NPP in patients with SCI. However, its conclusions are still inconsistent and there is no systematic review focusing on such issue. Therefore, this study will systematically discuss its efficacy and safety of BTTA for the treatment of NPP in patients with SCI, and will provide a systematic and comprehensive assessment for further research of NPP in patients with SCI. Its findings may provide helpful reference for both clinician and health-related policy makers.

### 3.1 Ethics and dissemination

This study will not need research ethics approval, because no confidential patient data will be used. We will publish this study on a peer-reviewed journal or through a conference presentation.

## Author contributions

**Conceptualization:** Zeng-mian Wang, Chun-jie Wei, Yao-jia Jiang.

**Data curation:** Zeng-mian Wang, Chun-jie Wei.

**Formal analysis:** Zeng-mian Wang, Ze-yu Wang.

**Funding acquisition:** Yao-jia Jiang.

**Investigation:** Yao-jia Jiang.

**Methodology:** Zeng-mian Wang, Chun-jie Wei.

**Project administration:** Yao-jia Jiang.

**Resources:** Zeng-mian Wang, Ze-yu Wang, Chun-jie Wei.

**Software:** Zeng-mian Wang, Ze-yu Wang, Chun-jie Wei.

**Supervision:** Yao-jia Jiang.

**Validation:** Zeng-mian Wang, Ze-yu Wang, Chun-jie Wei, Yao-jia Jiang.

**Visualization:** Zeng-mian Wang, Chun-jie Wei, Yao-jia Jiang.

**Writing – original draft:** Zeng-mian Wang, Ze-yu Wang, Yao-jia Jiang.

**Writing – review & editing:** Zeng-mian Wang, Ze-yu Wang, Chun-jie Wei, Yao-jia Jiang.
